# Supplementary material for: TUBB1 mutations cause thyroid dysgenesis associated with abnormal platelet physiology
Source: EMBO Mol Med. 2018 Nov 19;10(12):e9569. doi: 10.15252/emmm.201809569 (PMC6284387; doi:10.15252/emmm.201809569)

TUBB1 mutations cause thyroid dysgenesis associated with abnormal platelet physiology

Athanasia Stoupa, Frédéric Adam, Dulanjalee Kariyawasam, Catherine Strassel, Sanjay Gawade, Gabor Szinnai, Alexandre Kauskot, Dominique Lasne, Carsten Janke, Kathiresan Natarajan, Alain Schmitt, Christine Bole-Feysot, Patrick Nitschke, Juliane Léger, Fabienne Jabot-Hanin, Frédéric Tores, Anita Michel, Arnold Munnich, Claude Besmond, Raphaël Scharfmann, François Lanza, Delphine Borgel, Michel Polak and Aurore Carré

Appendix

Legends
Table S1
Table S2
Table S3
Figure S1
Figure S2
Figure S3
Figure S4
Figure S5

APPENDIX LEGENDS

Appendix Table S1

The table describes thyroid development in humans and mice, with thyroid expression markers at different stages (gestational weeks and embryonic days, respectively).

Appendix Table S2

Summary of morphological thyroid abnormalities in *Tubb1*$^{-/-}$ mice at each developmental stage. MA: median anlage, UB: ultimobranchial bodies

Appendix Table S3

Exact P-value for Figures 3-7

Appendix Fig S1

Whole exome sequencing data analysis for family F1 with variant filtering and the prioritisation strategy.

Appendix Fig S2

a - Tubb1 expression in sorted cells from mouse thyroid tissue at E17.5 and adulthood assessed using quantitative PCR: higher Tubb1 expression in platelets carrying the CD41 marker.

b - Top: immunofluorescence for β1-tubulin (in red), thyroglobulin (Tg, in green), and both merged (from left to right) in thyroid mouse tissue at E17.5 Bottom: immunofluorescence for β1-tubulin (in red), thyroglobulin (Tg, in green), and both merged (from left to right) in adult thyroid mouse tissue. Scale bar: 10μm

c - Immunofluorescence for β1-tubulin (in red), Thyroglobulin (Tg, in green), and magnification (from left to right) in thyroid mouse tissue at E17.5; Top: wild-type, Bottom *Tubb1*$^{-/-}$.

Appendix Fig S3

Western Blot of wild-type and mutants β1-tubulin proteins

Protein extracts (15ug) of transfected Nthy with wild-type or different mutants 48h post-tranfection.

a – western blotting with β1-tubulin antibody : expression of transfected and endogenous β1-tubulin in Nthy cells (wild-type β1-tubulin: 50KDa, GFP-wild-type β1-tubulin: 80KDa) β1-tubulin antibody recognizes the C-terminal region.

b - western blotting with GFP antibody : expression of transfected GFP-β1-tubulin in Nthy cells (GFP alone: 30KDa, GFP-106X β1-tubulin: 42KDa, GFP-35delG β1-tubulin: 32KDa).

c – western blotting with actin antibody

Appendix Fig S4

a - Expression of all β and α-tubulins in *Tubb1*$^{-/-}$ and wild-type thyroid tissue at E17.5 and adulthood. Note the increases in Tubb2a, Tubb2b, Tubb3 and Tubb5 and decreases in Tuba3, and Tuba4 at E17.5 in *Tubb1*$^{-/-}$ versus wild-type mice. β-tubulins were increased and α–tubulin decreases in adult *Tubb1*$^{-/-}$ versus wild-type mice (and in *Tubb1*$^{-/-}$ platelets studied by Schwer et al. (Schwer *et al*, 2001)) *$p<0.05$

b - Primer sequences for mouse α and β-tubulins used for quantitative PCR

Appendix Fig S5

Aggregation of washed platelets induced by ADP (5 or 10 µM) or collagen (0.6 and 0.8 µg/mL) was evaluated in three patients with thyroid ectopy treated by L-Thyroxine but not harboring *TUBB1* mutations. No increase of platelet aggregation was observed compared to controls (mean ± SEM of 5 controls).

Appendix

Table S1) Thyroid development in humans and in mice

Human in week	Mouse in Day	Development steps	Expression markers	
			Early steps	Late Steps
3GW	E8.5/E9.5	specification of thyroid progenitor cells, median anlage (MA)	Nkx2-1, Pax8, Foxe1	
4GW	E11.5	bud dislocation and migration of median anlage and ultimobranchial bodies (UB)	Nkx2-1, Pax8, Foxe1	
7GW	E13.5	end of migration, fusion of midline thyroid with UB and lobulation	Nkx2-1, Pax8, Foxe1, Calca	
10GW	E15.5	thyroid lobe enlargement, folliculogenesis	Nkx2-1, Pax8, Foxe1, Calca	Tg, Tpo, Tshr, Calca, CT
12GW	E17.5	terminal differentiation	Nkx2-1, Pax8, Foxe1, Calca	Tg, Tpo, Tshr, Calca, CT, T4

Appendix

Table S2) Morphological abnormalities of thyroids in *Tubb1*-/- mice

Embryonic stage	Structural features of the thyroid gland
E9.5	Abnormal shape in 2/5
E11.5	MA developmental delay in 3/6
E13.5	Fusion MA/UB delay in 3/4
E15.5	Pyramidal lobe in 1/4, holes throughout lobes
E17.5	Pyramidal lobe in 2/6, holes throughout lobes

Appendix Table S3

Exact *P*-value for Figures 3-7

**Figure 3B**	*P*
E13.5 vs E15.5	0.0012
E13.5 vs E17.5	0.0088
E13.5 vs Adult	0.0059

**Figure 3C**	*P*
Adult EpCam vs E17.5 Pecam	< 0,0001
Adult EpCam vs E17.5 CD45	0.0221
Adult EpCam vs Adult Pecam	0.0266
Adult EpCam vs Adult CD45	0.0148

**Figure 4B**		*P*
um2/g	E9.5 wt vs ko	0.0063
%Ki67/Nkx2-1 progenitors	wt vs ko	0.0317
um2	E13.5 wt vs ko	0.0085
	E15.5 wt vs ko	0.0173
	E17.5 wt vs ko	0.0223

**Figure 4C**		*P*
**E15.5 wt vs ko**	Tg	0.0155
	TPO	0.0201
	Tshr	0.0157
	Calca	0.0076
**E17.5 wt vs ko**	Foxe1	0.0191
	Pax8	0.0049
	Nkx2-1	0.0346
	Tg	0.0051
	Calca	0.0012

**Figure 4D**	*P*
% T4 vs thyroid area, wt vs ko	0.0004

**Figure 5A**	*P*
T4 wt vs ko	0.0025

**Figure 5D**		*P*
QPCR wt vs ko	Chop	0.0105
	xbps	0.0139
western blot wt vs ko	Chop	0.0471

**Figure 6C**		*P*
shaft thickness	P1	0,0103
	P4	<0.001
Coiled element diameter	P1	0,0106
	P4	<0.001

**Figure 7A**		*P*
TUBB1	P1	<0.001
	P3	<0.001
	P4	<0.001
	P6	<0.001
	P7	<0.001

**Figure 7B**		*P*
PAC-1 (%)	P1	<0.001
	P3	<0.001
PAC-1 (MFI)	P1	0.03
	P3	0.0062

**Figure 7C**		*P*
ADP	P1 5uM	<0.001
	P3 5uM	0.0495
	P1 10uM	<0.001
	P3 10uM	0.0061
Collagen	P1 0.6	<0.001
	P1 0.8	0.0105

Appendix

Figure S1) WES data analysis for family 1

Whole set of variants according to familial mode analysis

Substitutions, n=13,5391
Deletions, n=8,625
Insertions, n=7,438

Genes, n=22,790

Variants Filtering and Prioritization strategy

Filters:

- **Consequence of the variation: missense, non-synonymous, frameshift, stop-gained, codon-stop, splicing**
- **Type of variation: substitution, deletion, insertion**
- **Public databases (dbSNP, EVS, 1,000 genomes, ExAC) filtering: frequency ≦1%**
- **In-house databases filtering: already-seen (« déjà-vu ») variants : ≦ 5 among 11,811 WES**
- **In silico (SIFT, Polyphen-2) predictions: all status, all CADD scores**

Substitutions, n=429
Deletions, n=14
Insertions, n=13

Genes, n=446

Variants Filtering according to Transmission mode

Recessif

Substitutions, n=1
Del/Ins: 0
Genes n=1, ***TUBB1***

De novo

Substitutions, n=3
Del/Ins: 0
Genes, n=3

Compound

Sub/Del/Ins, n=0
Genes, n=0

Assessment of relevant variants

- **Gene/mutation (HGMD, Clinical variants)**
- **Clinical input vs phenotype/ mode of inheritance**
- **Data from literature research**
- **Sanger sequencing and familial segregation**

Substitutions, n=1

Genes n=1, ***TUBB1***

Appendix

Figure S2a) Tubb1 expression in sorted cells from thyroid tissue

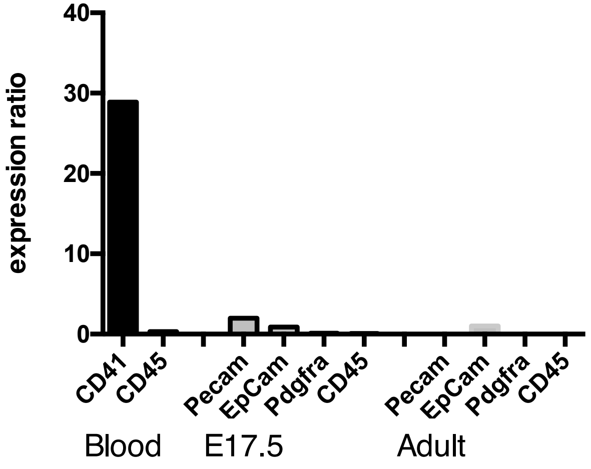

Figure S2b) Tubb1 by immunohistochemistry in mouse thyroid tissue

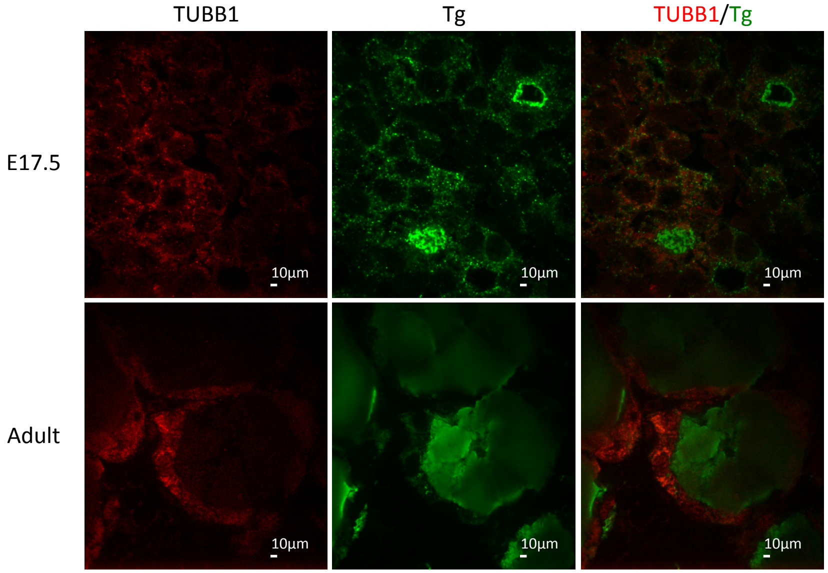

Figure S2c) Tubb1 by immunohistochemistry in wild-type and *Tubb1-/-* mouse thyroid tissue

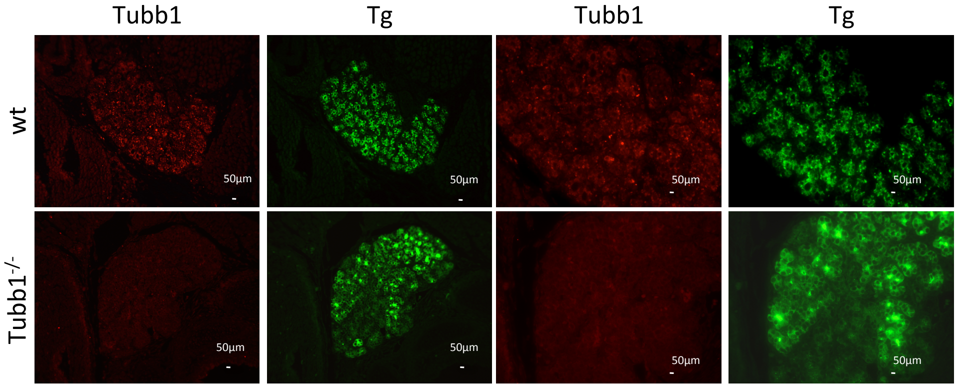

Appendix

Figure S3) Western Blot of wild-type and mutants β1-tubulin proteins

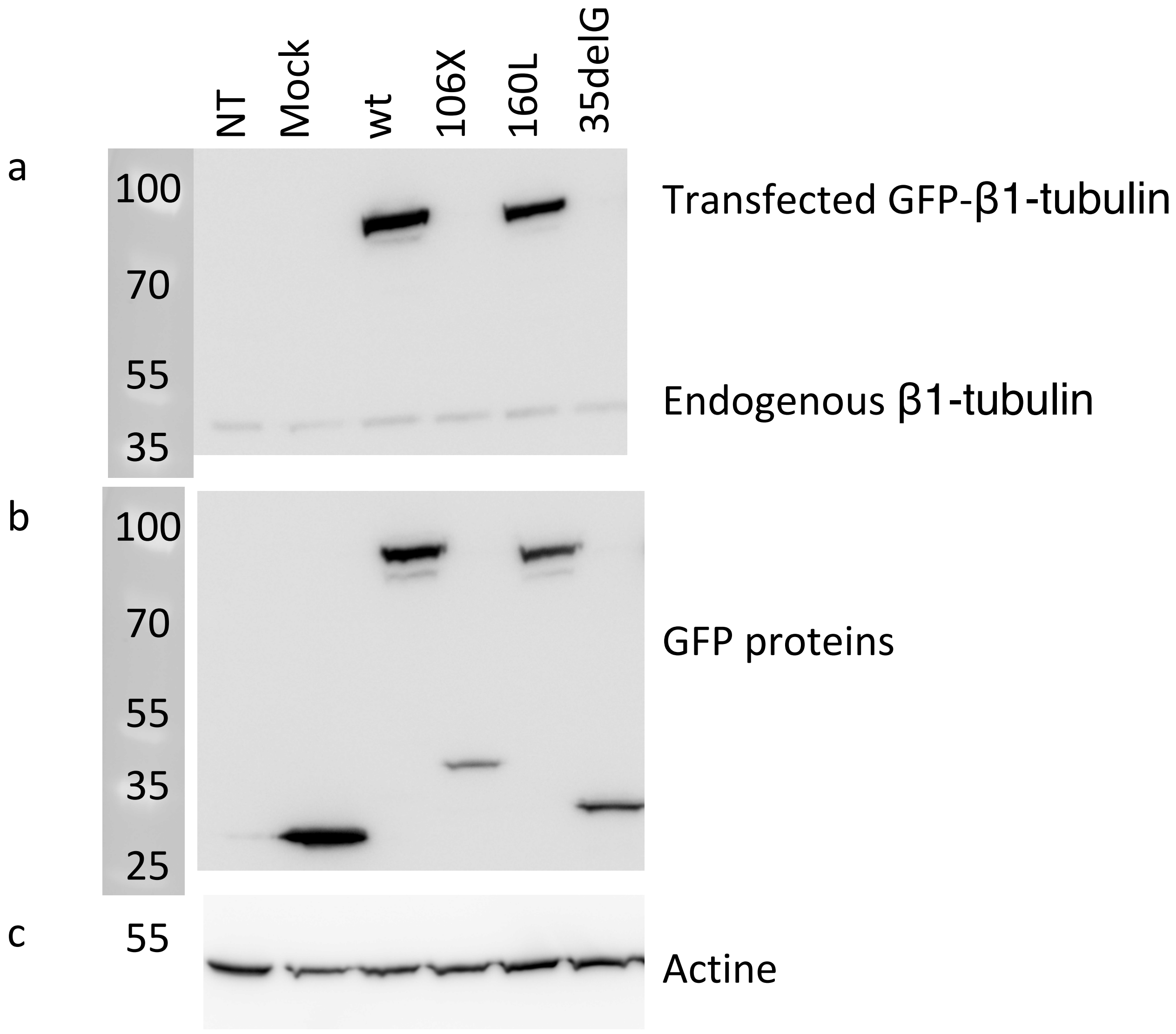

Appendix

Figure S4) Tubulins alpha and beta expression

a) Expression of beta and alpha tubulins in thyroid tissue from *Tubb1*-/- vs. wild-type mice

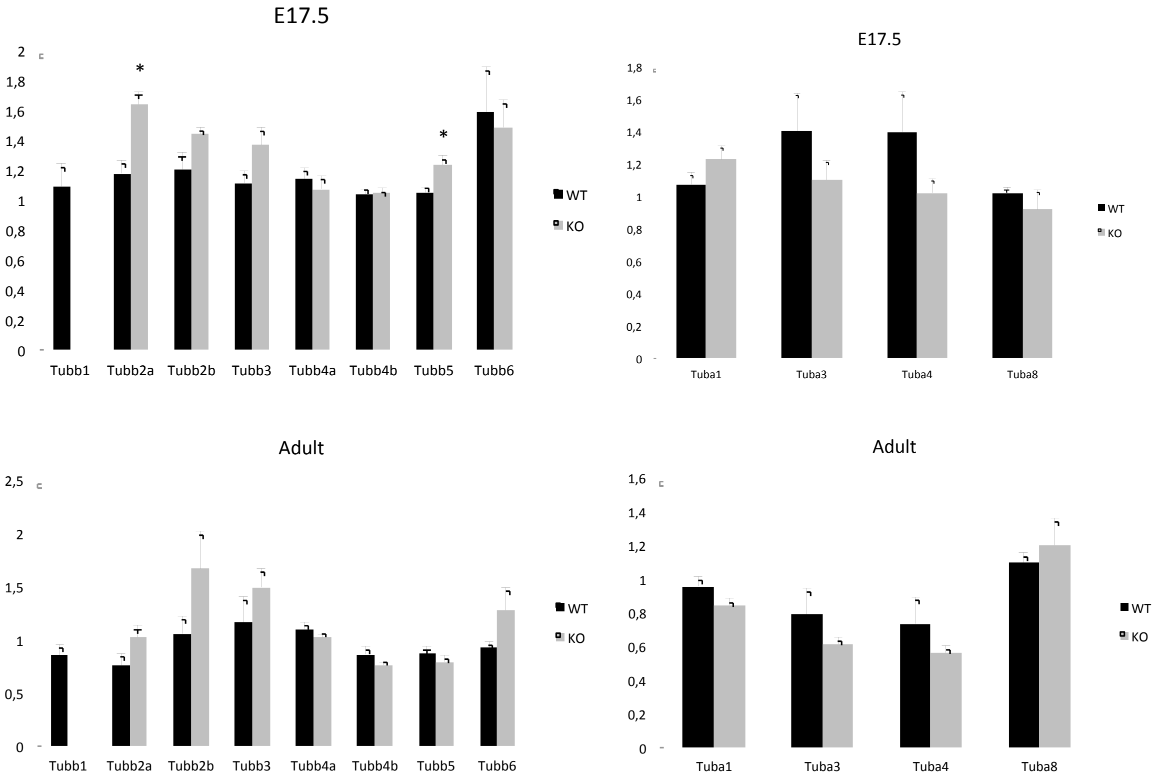

b) Primer sequences for alpha and beta tubulins

Gene name	Accession number	Forward primera	Reverse primer	Protein length (pb)
TubB1	NM_001080971	TCCAGCAGATTGGGAGTCCTCTTT	ATGCACGATCTGGAAACCCTGTAG	180
TubB2a	NM_009450	CTCAGTGAGGTCGGGACCATT	GTGGCCTTTTGCCCAGTTATTT	94
TubB2b	NM_023716	ATTCAGGCGGGCCAGTGCGGCAA	GGTCTATACCATGCTCATCA	76
TubB3	NM_023279	CCTTTGGACACCTATTCAGGCCCGA	CGACATCTAGGACTGAGTCCACC	120
TubB4a	NM_009451	CGGCACCATGGACTCTGTCCGCT	CCAGGACGGCATCCACTAACT	143
TuB4b	NM_146116	GATCTTCAGACCTGATAACTT	ACCAGCTCTGCACCTTCTGTGTA	90
TubB5	NM_011655	CGTTCGCTCAGGTCCTTTTGGC	AACTCAGCTCCCTCTGTGTAGTGGC	111
Tubb6	NM_026473	GGGCCTTTTGGGCAACTCTTCCG	AATCCACCAGCTCCGCGCCCTCCGT	109
TubA1	NM_011653/NM_011654	CAGTGTTCGTAGACCTGGAACC	TCCTCCTTGCCTGTGATGAGCTGC	100
TubA3	NM_009446/NM_009449	ACAAAGCGCACCATCCAGTT	CACTTTGGCCAGGTCTCCCC	105
TubA4	NM_009447	TACACCATTGGCAAGGAGATCATCG	AAAGCTGTGGAATACTAGGAA	102
TubA8	NM_017379	ACTATACGGTGGGCAAGGAGAG	TCAGCAGAGAAGTAAAGCCAGAT	141

Appendix

Figure S5) Normal platelet aggregations in patients without *TUBB1* mutations but treated by L-Thyroxine

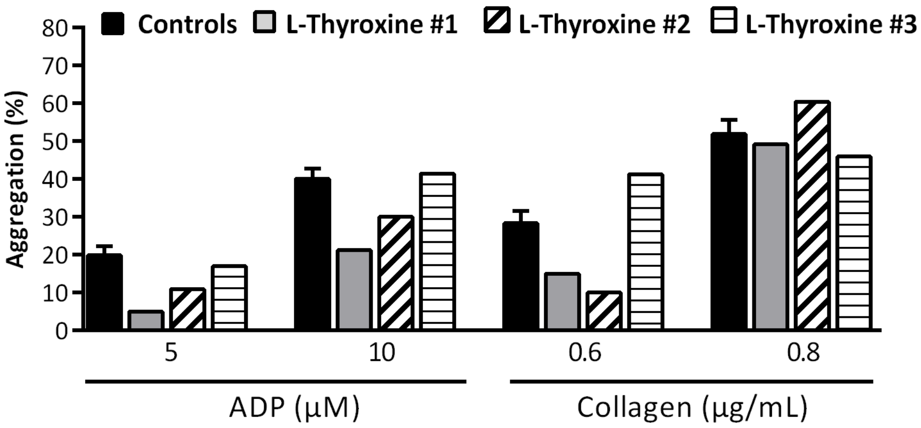

Supplement: Supplementary file 1 — Appendix [file EMMM-10-e9569-s001.pdf]
