# Supplementary material for: TUBB1 mutations cause thyroid dysgenesis associated with abnormal platelet physiology
Source: EMBO Mol Med. 2018 Nov 19;10(12):e9569. doi: 10.15252/emmm.201809569 (PMC6284387; doi:10.15252/emmm.201809569)
Supplement: Supplementary file 2 — Table EV1 [file EMMM-10-e9569-s002.pdf]

Table EV1 List of rare functional variants found in *TUBB1*

chr	pos	dbSNP_name	ref	alt	snpEff_type	MAF in EXAC (r0.2)	MAF in EXAC (r0.3)	Nb of Het cases	Nb of Homozyg cases	Nb of Het Controls	Nb of Homozyg controls
20	57594584	rs45599044	G	T	stop_gained	0	0	0	0	1	0
20	57594612	rs77324804	G	-	frameshift_variant	3,25E-05	2,47E-05	1	0	0	0
20	57597910	.	T	G	missense_variant	8,17E-06	8,27E-06	0	0	1	0
20	57598800	.	C	G	stop_gained	0	0	1	0	0	0
20	57598808	rs41303899	G	A	missense_variant	8,78E-04	8,73E-04	2	0	2	0
20	57598961	rs759117911	C	T	missense_variant	8,13E-06	8,24E-06	0	1	0	0
20	57599401	rs62639974	C	T	missense_variant	4,46E-03	4,43E-03	8	0	3	1
20	57599527	rs115253190	G	A	missense_variant	2,18E-03	2,17E-03	1	0	0	0
