# Supplementary material for: TUBB1 mutations cause thyroid dysgenesis associated with abnormal platelet physiology
Source: EMBO Mol Med. 2018 Nov 19;10(12):e9569. doi: 10.15252/emmm.201809569 (PMC6284387; doi:10.15252/emmm.201809569)
Supplement: Supplementary file 3 — Table EV2 [file EMMM-10-e9569-s003.docx]

**Table EV2** Primers list of human TUBB1 gene for Sanger sequencing

| TUBB1 e1F | AGATGGACAGGGAAAGCCCTTG |
| --- | --- |
| TUBB1 e1R | GCTAGACTTATTGTCTTCCCTGCTAAC |
| TUBB1 e2F | AAGGCCCTAAAAGAATGTCTTGG |
| TUBB1 e2R | GCCTCCCTGAGCATAGACATCACT |
| TUBB1 e3-4aF | CCCTTCTAGAATATCCATGATTTT |
| TUBB1 e3-4aR | TCAGCTGGTGGATAGACAGAAC |
| TUBB1 e4bF | CGAGCTGATCGAGAATGTCCTA |
| TUBB1 e4bR | TGGACATCTTGCCCCGGAAAATGC |
| TUBB1 e4cF | TGTTCGATGCCCGCAATACC |
| TUBB1 e4cR | TTTTGGAGTGCTGCAGGAGA |
