# Supplementary figures and images for: TUBB1 mutations cause thyroid dysgenesis associated with abnormal platelet physiology
Source: EMBO Mol Med. 2018 Nov 19;10(12):e9569. doi: 10.15252/emmm.201809569 (PMC6284387; doi:10.15252/emmm.201809569)

Fig 5D

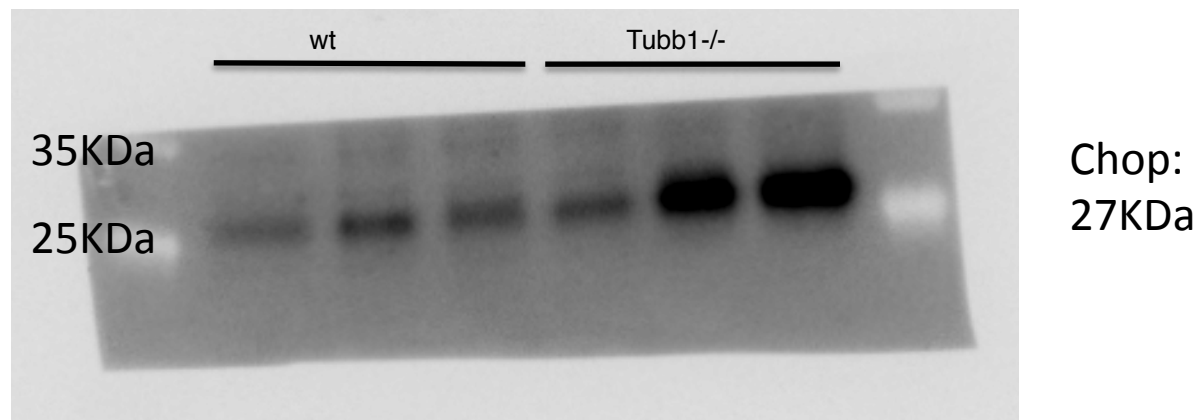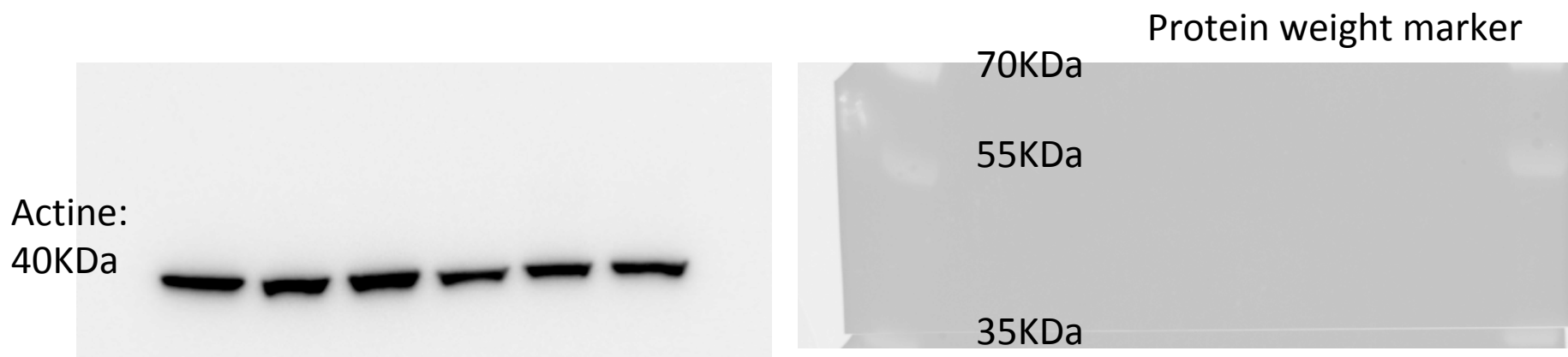

Supplement: Supplementary file 5 — Source Data for Figure 5 [file EMMM-10-e9569-s004.pdf]
